# The impact of social participation on the health status of the older adult: An empirical study based on CGSS 2021 data

**DOI:** 10.1371/journal.pone.0305820

**Published:** 2024-06-25

**Authors:** Zebo Zhang, Lei Jin, Jiajun Liu, Dongling Liao, Xiaolin Zhang

**Affiliations:** School of Physical Education and Health, Guangxi Normal University, Guilin, Guangxi, China; University of Environment and Sustainable Development, GHANA

## Abstract

**Introduction:**

With the rapid pace of population aging, the health issues of the older adult have garnered widespread attention. Social participation plays a pivotal role in the health of the older adult. This study aims to explore the impact of social participation on the health status of the older adult.

**Methods:**

Using a binary logistic regression model, this study analyzes the influence of social participation methods on the health status of older adult individuals in China based on cross-sectional data from the "China Comprehensive Social Survey" in 2021. The study sample comprises individuals aged 60 to 99 years.

**Results:**

It was found that participation in physical activities [P<0.001, OR = 1.907], social and recreational activities [P<0.001, OR = 1.387], and online activities [P<0.001, OR = 1.808] were significantly positively correlated with the health status of the older adult.

**Conclusions:**

The health of older adults is influenced by a combination of physical activities, social and recreational activities, and online activities. Good health is closely associated with high levels of physical activity. Engaging in physical exercise promotes physiological health, while participating in social and recreational activities has a significant impact on cognitive and depressive states. Additionally, involvement in online activities helps alleviate feelings of loneliness and enhances overall well-being.

**Recommendations:**

1)Promote the development of physical activities for the older adult: Create an integrated environment for physical exercise. 2)Expand the social circle of the older adult: Construct diverse and structured communities to enhance well-being. 3)Develop online activities for the older adult: Facilitate their integration into the digital age. 4)Foster interdisciplinary collaboration for older adult health: Build partnerships across various domains to promote older adult health.

## Introduction

According to the World Health Organization’s report in 2020, the global population aged 60 and above has surpassed the number of children aged 5 and below. By 2030, one-sixth of the world’s population will be aged 60 or above, increasing from 1 billion in 2020 to 1.4 billion. The global population aged 60 and above is projected to double by 2050, reaching 2.1 billion. During the period from 2020 to 2050, it is expected that the number of individuals aged 80 and above will double, reaching 426 million. This unprecedented growth is set to accelerate in the coming decades, particularly in developing countries [1]. As the world experiences population aging, aging has become a common challenge faced by societies today. The international response has evolved from the concept of "successful aging" to "healthy aging" and further to "active aging." The role of the older adult is transitioning from being "passive dependents" to "active participants in societal activities" [2–4]. Increasing social participation among the older adult to enhance health and well-being has become a clear policy goal for many national governments and the European Commission. Numerous scholars have conducted extensive research on the health status of older adults. However, there has been relatively less research on the impact of social participation on the health status of older adults. In order to more effectively understand the influencing factors of the health status of older adults and the relationship between social participation and health status,empirical research was conducted using data from the China General Social Survey in 2021. This study specifically explored the correlations between physical activities, social and entertainment activities, online activities, and the health status of the older adult. The findings from this empirical investigation are accompanied by corresponding policy recommendations, offering theoretical insights to promote the health of the older adult.

### Literature review

Social participation is an interdisciplinary academic concept that lacks a universally accepted, precise definition. Research on factors influencing older adult health has predominantly focused on economic and individual attributes [5]. However, the impact of social participation on the physical and mental well-being of the older adult is profound, making it a focal point of interdisciplinary studies in demographics, sociology, psychology, and other fields [6]. The core idea of older adult social participation revolves around the deep interaction between older adult individuals and multi-level social ecological systems [7]. In China, the emphasis on older adult social participation underscores achieving the "independence, involvement, care, self-fulfillment, and dignity" of the aging population. It encourages older adult individuals of all ages and health statuses to engage in socialist material and spiritual civilization innovations and multidimensional creations [8]. Three related concepts of social participation include social connections, informal social involvement, and volunteer service [9]. In practice, this encompasses interactions with family and friends, self-perceived support, trust, volunteer service, civic and political engagement, and the use of information and communication technologies [10]. The World Health Organization, in 2021, defined social participation as a policy direction to promote health equity. It is seen as an empowering process that enhances the inclusive governance of civil society and fosters active societal roles during the course of achieving universal health coverage [11]. Social participation is considered a crucial component of "active aging" and "healthy aging," serving as a key means to enhance the well-being of the older adult and address the challenges of population aging [12,13]. Older adult individuals need to develop self-awareness through engagement in social activities, as clearer self-awareness contributes to maintaining vitality in life [7]. Social participation has a significant impact on the physical and mental health of the older adult [14]. Social activity involvement forms the foundation of older adult social participation [15], and social relationships have a positive influence on health [16]. Physical exercise, as a vital means for older adult individuals to maintain health, plays a positive role in disease prevention, slowing functional decline, enhancing physical fitness, and improving psychological well-being [17]. Engaging in social activities helps expand social networks, increase social support, reduce feelings of loneliness, and ultimately enhance the quality of life [18]. Therefore, promoting social participation among the older adult and creating effective participation platforms may contribute to maintaining their physical and mental health [19].

In summary, previous domestic and international research has laid a robust theoretical foundation for this paper, providing objective and scientific support in the selection of control variables. However, upon a meticulous examination of the existing literature, certain shortcomings in prior studies have been identified. Firstly, there is an insufficiently comprehensive selection of social participation modes. Secondly, due to variations in national contexts, scholars tend to prioritize variables based on their own country’s circumstances. Thirdly, there is a limited number of scholarly articles within the academic community specifically investigating the impact of online activities on the health of the older adult. Addressing these gaps, this paper utilizes data from the China General Social Survey (CGSS) in 2021 to analyze the influence of various social participation modes on the health of the older adult. Specifically, it explores the impact coefficients of physical exercise, social and recreational activities, and online activities within the spectrum of social participation modes on the health of older adult individuals. The objective is to offer new evidence regarding the significance of diversity in social participation modes on the health of the older adult, thereby contributing novel insights to the discourse on active aging.

### Propose the hypothesis

Proposed Hypothesis 1: Engagement in sports activities significantly influences the health of the older adult.

Proposed Hypothesis 2: Participation in social and recreational activities significantly affects the health of the older adult.

Proposed Hypothesis 3: Involvement in online activities has a significant impact on the health of the older adult.

## Method

### Data source

This study utilizes data from the Chinese General Social Survey (CGSS) conducted in 2021. The CGSS, a collaborative effort involving Renmin University of China and academic institutions nationwide, represents the first nationwide, comprehensive, and continuous large-scale social survey project in China. Initiated in 2003, the survey is conducted annually, systematically collecting data on various aspects of Chinese individuals and society. It aims to capture long-term trends in social changes, explore socially significant theoretical and practical issues, promote openness and sharing in domestic social science research, and provide data for governmental decision-making and international comparative studies. The CGSS employs a rigorous scientific sampling approach, utilizing a multi-stage, stratified random sampling method to survey over ten thousand households nationwide each year. The sampling covers urban and rural households in all 31 provinces, autonomous regions, and directly administered municipalities of China. Data is collected at multiple levels, including social, community, family, and individual levels. The content related to the older adult encompasses information such as personal demographics, health status, lifestyle, and social security. This study investigates the impact of social participation modes on the health status of the older adult. To meet the research requirements, target data were screened, ineffective and missing values in key variables were eliminated, resulting in a final sample size of 2,880. Subsequently, a logistic regression model was established based on this sample to analyze the correlation and relevance between social participation modes and the health status of the older adult.

### Model setting

The primary objective of this study is to examine the relationships between the health status of the older adult and various variables. The dependent variable is the "health status of the older adult," which has only two possible outcomes: "healthy" and "unhealthy." It represents a binary categorical variable, and thus, an analysis is conducted using a binary logistic regression model [20]. If the health status of the older adult is classified as healthy, it is denoted as "1"; conversely, if it is unhealthy, it is denoted as "0." The basic form of the model is expressed as follows (Formula 1):

Then the probability of the health status of the older adult is (Formula 2):


$$Ln(\frac{p(y=1)}{1-p(y=1)})=\alpha +\overset{k}{\underset{i=1}{\sum }}\beta_{i}x_{i}$$

$$p(y=1|x)=\frac{e^{\alpha +\sum_{i=1}^{k}\beta_{i}x_{i}}}{1+e^{\alpha +\sum_{i=1}^{k}\beta_{i}x_{i}}}$$

In Formulas (1) and (2), X represents the ith explanatory variable influencing the health status of the older adult, k is the number of explanatory variables, α is the intercept term, and β_i_ denotes the coefficient associated with the ith explanatory variable. The coefficient reflects both the direction and magnitude of the impact of each variable on the health status of the older adult. The probability of the health status of the older adult relative to the probability of being unhealthy is expressed as the odds ratio. e^βi^ The odds ratio reflects the multiplicative change in the odds of the event occurring for every one-unit change in the explanatory variable.

### Credit and validity test

The analysis of the CGSS 2021 survey questionnaire data in this study was conducted using IBM SPSS 26 software. The data underwent tests for reliability and validity, with the study employing Cronbach’s alpha coefficient, KMO (Kaiser-Meyer-Olkin) test, and Bartlett’s sphericity test. A reliability index ranging from 0.65 to 0.70 is considered the minimum specified value, 0.70 to 0.80 is considered adequate, and 0.80 to 0.90 is considered very good. For the KMO test, a coefficient greater than 0.5, with a significance level (P) less than 0.05, indicates structural validity of the questionnaire. The reliability and validity tests were conducted on questionnaire A30 using IBM SPSS 26. The Cronbach’s Alpha result for the reliability test was 0.793, with a standardized item’s Cronbach’s α of 0.797. With 12 items, the reliability index falls within the 0.70 to 0.80 range, indicating high reliability of the questionnaire. The KMO value, at 0.83, is greater than 0.5, suggesting that the data from this questionnaire are suitable for principal component analysis. A higher KMO value approaching 1 indicates stronger correlations among the factor indicators. The Bartlett’s sphericity test yielded a P-value less than 0.0001, signifying that the data are mutually independent and follow a spherical distribution. Considering the results of these tests collectively, the overall reliability and validity of the survey questionnaire in this study are deemed satisfactory, supporting its suitability for investigating the impact of social participation modes on the health status of the older adult (Table 1).

**Table 1:** Questionnaire reliability and validity test.

Test Methods		Parameter name	Parameter	
Reliability Statistics		Cronbach’s Alpha	0.793	
		Cronbach’s Alpha Based on Standardized Items	0.797	
		N of Items	12	
Factor analysis	KMO and Bartlett’s Test	Kaiser-Meyer-Olkin Measure of Sampling Adequacy.	0.830	
		Bartlett’s Test of Sphericity	Approx. Chi-Square	2.139E4
			df	66
			Sig.	0.000

Note: sig = 0.000, indicating p < 0.05.

### Variable selection

#### Dependent variable

The dependent variable in this paper is the health status of the older adult. We assessed the health status based on responses to the question in the CGSS 2021 survey questionnaire: "How do you perceive your current physical health status?" The corresponding code for this question is "A15." Responses categorized as "very unhealthy" or"somewhat unhealthy" are considered as indicating an unhealthy condition, assigning a value of "0" to represent the older adult person’s health as unhealthy. Responses categorized as "fair," "somewhat healthy," or "very healthy" are considered as indicating a healthy condition, and a value of "1" is assigned to represent the older adult person’s health as healthy. Responses such as "don’t know," "refuse to answer," and missing values are excluded from this measurement to ensure the accuracy of the results.

#### Independent variables

Social participation modes are defined as the ways in which older adults select activities in the social environment for the purpose of interaction and value exchange. Physical activity, as a primary form of activity, aims to improve health, enhance physical fitness, exercise the body, promote social interaction, and strengthen mental well-being. Social recreational activities, on the other hand, focus on social interaction and entertainment, aiming to foster interpersonal relationships, enhance community cohesion, increase personal happiness, and enjoy the pleasures of life. Internet activities encompass various activities conducted through the internet, including but not limited to online socializing, participation in virtual communities, digital entertainment, distance learning, digital skills training, online gaming, and digital art creation. This study selects physical activity, social recreational activities, and internet activities as independent variables in the model to investigate social participation modes.

Independent Variable 1 (Physical Exercise), Derived from the CGSS 2021 questionnaire, this variable (A30) pertains to the question: "In the past year, how often did you engage in the following activities during your leisure time?" Specifically, we focus on response option 9: "Participating in physical exercise." Responses indicating regular participation in physical exercise, such as "every day," "several times a week," "several times a month," and "several times a year or less," are assigned a value of "1." Conversely, responses indicating no participation in physical exercise, such as "never," are assigned a value of "0." Responses categorized as "don’t know," "refuse to answer," and missing values are excluded from the analysis.

Independent Variable 2(Social and Recreational Activities), Derived from the CGSS 2021 questionnaire, this variable (A31a) corresponds to the question: "How often do you engage in social and recreational activities with your neighbors (such as visiting each other, watching TV together, having meals, playing cards, etc.)?" Responses indicating frequent engagement in social and recreational activities, such as "almost every day," "1 to 2 times a week," "several times a month," "about once a month," "several times a year," and "once a year or less," are assigned a value of "1" to represent participation in social and recreational activities. Conversely, responses indicating no participation in social and recreational activities, such as "never," are assigned a value of "0." Responses categorized as "don’t know," "refuse to answer," and missing values are excluded from the analysis.

Independent Variable 3(Online Activities), Derived from the CGSS 2021 questionnaire, this variable (A30) corresponds to the question: "In the past year, how often did you engage in the following activities during your leisure time?" Specifically, we focus on response option 12: "Using the internet." Responses indicating regular engagement in online activities, such as "every day," "several times a week," "several times a month," and "several times a year or less," are assigned a value of "1" to represent participation in online activities. Conversely, responses indicating no participation in online activities, such as "never," are assigned a value of "0." Responses categorized as "don’t know," "refuse to answer," and missing values are excluded from the analysis. Descriptive analysis of the relevant independent variables is presented in Table 2.

**Table 2:** Descriptive statistical analysis of the relevant independent variables(N = 2880).

Variables	Illustrate	Assignment	Frequency	Standard error
Sports Activities	Engages in Sports Activities	1	0.537	0.009
	Does not engage in Sports Activities	0	0.463	
Social and Entertainment Activities	Engages in Social and Entertainment Activities	1	0.715	0.008
	Does not engage in Social and Entertainment Activities	0	0.285	
Online Activities	Engages in Online Activities	1	0.350	0.009
	Does not engage in Online Activities	0	0.650	

### Control variables

Based on an extensive literature review and considering the specific focus of our research, we have identified the following six control variables: 1) Gender, coded as a dummy variable (Male = "1"; Female = "2"); 2) Age, recoded from the birthdate variable as a dummy variable (1961–1941 = "1"; 1940–1922 = "2"); 3) Household registration type, recoded as a dummy variable (Agricultural household registration = "1"; Resident household registration = "2"; Other = "3"); 4) Education level, recoded as a dummy variable (Primary school and below = "1"; Secondary school = "2"; Junior college = "3"; Bachelor’s degree = "4"; Graduate and above = "5"); 5) Marital status, recoded as a dummy variable (Unmarried = "1"; Married = "2"; Widowed = "3"); 6) Type of occupation, recoded as a dummy variable (Agricultural work = "1"; Non-agricultural work = "2"; Unemployed = "3"). Responses of "don’t know," "refuse to answer," and missing values for these six variables are excluded. Descriptive analysis of these control variables is presented in Table 3.

**Table 3:** Descriptive analysis of the relevant control variables(N = 2880).

Variables	Illustrate	Assignment	Frequency	Standard error
Gender	male	1	0.488	0.009
	female	2	0.512	
Age	60–80	1	0.912	0.005
	81–99	2	0.088	
Affiliation	Agricultural account	1	0.583	0.015
	Residents registered permanent Residence	2	0.233	
	Other household registration	3	0.184	
Education level	Primary School and Below	1	0.535	0.012
	Middle School	2	0.418	
	College Diploma	3	0.025	
	Undergraduate Degree	4	0.020	
	Graduate and Above	5	0.001	
Marital Status	Unmarried	1	0.012	0.008
	Married	2	0.739	
	Widowed	3	0.249	
Occupation Type	Agricultural	1	0.193	0.015
	Non-agricultural	2	0.066	
	Unemployed	3	0.741	

## Results

This study investigates the influence of social participation on the health status of the older adult, utilizing logistic regression models to specifically explore how the independent variables, namely sports activities, social entertainment activities, and online activities, affect the dependent variable—the health status of the older adult. Based on an extensive literature review from demographic and sociological perspectives, six variables—gender, age, household registration type, education level, marital status, and type of occupation—are chosen as control variables. Four regression models are established using logistic regression, and the overall data for Models 1, 2, 3, and 4 are presented in Table 4. The logistic regression results are reflected in Table 5, indicating a prediction accuracy of 69% for all four models. The Omnibus Tests of Model Coefficients (P<0.005) confirm that at least one independent variable in Models 1, 2, 3, and 4 has a significantly non-zero regression coefficient, establishing the overall statistical significance of the models. The significance test (P<0.005) demonstrates a significant linear relationship between the independent variables and the dependent variable in each model. The comprehensive evaluation of the four models, considering the -2 times log-likelihood values, Cox & Snell R2, Nagelkerke R2, likelihood ratio chi-square values, and degrees of freedom, indicates a good fit for analysis results.

**Table 4:** Overall test results of the model for factors influencing the health status of the older adult(N = 2880).

	Prediction accuracy (%)	− 2 times the log likelihood value	Cox&Snell R^2^	Nagelkerke R^2^	Chi-square	df	Omnibus Tests of Model Coefficients (sig)
Model 1	69	3413.426^a^	0.052	0.073	152.914	6	0.000
Model 2	68.8	3357.853^a^	0.070	0.098	208.487	7	0.000
Model 2	69.2	3400.786^a^	0.056	0.079	165.554	7	0.000
Model 4	69.1	3378.164^a^	0.063	0.089	188.176	7	0.000

**Table 5:** Regression analysis of the impact of social participation modes on the health status of the older adult(N = 2880).

	Model 1			Model 2		
	Regression coefficient	Standard error	OR	Regression coefficient	Standard error	OR
Constants	0.555	0.304	1.742	0.351	0.308	1.420
Gender	-0.175	0.087	0.839	-0.150	0.088	0.860
Age	-0.315	0.147	0.730	-0.226	0.148	0.798
Affiliation	0.452	0.066	1.572	0.388	0.067	1.474
Education level	0.432	0.080	1.541	0.353	0.081	1.424
Marital Status	-0.066	0.092	0.936	-0.044	0.094	0.957
Occupation Type	-0.130	0.054	0.879	-0.163	0.055	0.850
Sports Activities				0.646	0.087	1.907
	Model 3			Model 4		
	Regression coefficient	Standard error	OR	Regression coefficient	Standard error	OR
Constants	0.295	0.313	1.343	0.530	0.305	1.698
Gender	-0.179	0.087	0.836	-0.192	0.088	0.826
Age	-0.302	0.147	0.739	-0.207	0.148	0.813
Affiliation	0.470	0.066	1.600	0.401	0.066	1.494
Education level	0.443	0.080	1.558	0.298	0.082	1.347
Marital Status	-0.082	0.093	0.921	-0.040	0.093	0.961
Occupation Type	-0.124	0.055	0.884	-0.141	0.055	0.868
Social and Entertainment Activities	0.327	0.092	1.387			
Online Activities				0.592	0.101	1.808

Note: * indicates significant at the 10% level

** indicates significant at the 5% level

***indicates significant at the 1% level.

### Model 1: The impact of control variables on the health status of the older adult

Model 1 illustrates the influence of control variables (gender, age, household registration type, current education level, marital status, and type of occupation) on the health status of the older adult, where β represents the regression coefficients. Our initial focus is on examining the impact of control variables on the dependent variable (health status). Through model analysis, it is observed that the selected control variables significantly affect the health status of the older adult. Specifically, household registration type and current education level exhibit a positive correlation. This suggests that older adult individuals from different regions or household registration types are more likely to access medical resources, social support, or other factors beneficial to health. Additionally, those with higher education levels are more inclined to adopt health-promoting behaviors, possess better health knowledge and awareness, or have received improved health protection throughout their professional careers. Gender, age, marital status, and type of occupation are negatively correlated with health status, gender may exhibit variations in certain health indicators. With advancing age, individuals’ health status may gradually decline, reflecting a common trend among the older adult population where physiological functions and immune systems gradually deteriorate, leading to an increase in health problems. Additionally, marital status is also associated with health status, with married individuals exhibiting better health outcomes compared to unmarried or divorced individuals. Lastly, the negative correlation between type of occupation and health status implies that certain professions or work environments have adverse effects on individual health. For instance, some occupations may expose individuals to harmful substances or high-stress environments, thereby leading to the occurrence or exacerbation of health issues.

### Model 2: The impact of participating in physical activities on the health status of the older adult

Model 2 extends Model 1 by incorporating the independent variable 1 (physical activities). The significance of the control variables remains largely unchanged. The regression coefficient of independent variable 1 (physical activities) is 0.646, surpassing the regression coefficients of the control variables. With an OR value of 1.907, this indicates a more pronounced influence of physical activities on the health status of the older adult when other factors remain constant. An OR value of 1.907 signifies that for each additional unit of engagement in physical activities, the odds of positive health status among the older adult increase by a factor of 1.907. This underscores a positive correlation between physical activities and the health status of the older adult.

### Model 3: The impact of participating in social and recreational activities on the health status of the older adult

Model 3 builds upon Model 1 by introducing independent variable 2 (social and recreational activities). The significance of the control variables remains relatively stable. The regression coefficient for independent variable 2 (social and recreational activities) is 0.327, comparable to the regression coefficients of the control variables. With an OR value of 1.387, it indicates a relatively modest impact of social and recreational activities on the health status of the older adult. Furthermore, the influence is akin to that of the control variables. While social and recreational activities exhibit a positive impact on the health status of the older adult, the effect is relatively modest.

### Model 4: The impact of engaging in online activities on the health status of the older adult

Model 4 extends Model 1 by introducing independent variable 3 (online activities). The significance of the control variables remains relatively consistent. The regression coefficient for independent variable 1 (physical activities) is 0.592, surpassing the coefficients of the control variables, with an OR of 1.808. This suggests that for each additional unit of physical activity, the odds of positive health outcomes for the older adult increase by a factor of 1.808. The results from this model indicate that physical activities have a relatively substantial impact on the health status of the older adult, and this impact is more pronounced than that of the control variables. With an OR greater than 1, it signifies a positive influence of physical activities on the health status of the older adult, demonstrating a relatively strong effect.

In summary, the analysis reveals significant influences of social participation modes—participation in physical activities, social and recreational activities, and online activities—on the health status of the older adult. The hypotheses proposed in the article are validated: Hypothesis 1—Engaging in physical activities significantly impacts the health of the older adult. Hypothesis 2—Engaging in social and recreational activities significantly affects the health of the older adult. Hypothesis 3—Engaging in online activities significantly influences the health of the older adult. It is noteworthy that the impact of engaging in social and recreational activities on the health status of the older adult is comparatively smaller than that of independent variables 1 (physical activities) and 3 (online activities).

## Discussion and policy recommendations

First, exercise can serve as an intervention to alleviate loneliness [21], benefiting the psychological well-being of older adults. Moreover, older adults who engage in physical exercise exhibit significantly better physiological, psychological, and social health, as well as overall health scores and physical condition compared to non-exercisers [22]. The paper highlights the significant impact of older adults’ participation in sports activities on physical health. Actively engaging in sports activities and exercise is conducive to improving the physical health of older individuals. However, the current sports environment is more suitable for younger people, and the facilities for older adult sports are lacking. To address this issue, we propose the construction of integrated facilities for older adult sports exercise through government policies. This initiative aims to create a holistic sports environment encompassing facilities, promotion, activities, personnel, and incentives, thereby enhancing older adult participation in sports and promoting their physical health:1) The government can invest in building community sports facilities tailored to the older adult, such as walking paths and fitness equipment areas, to provide convenient exercise venues. 2) Support social organizations in conducting regular health promotion activities, such as health lectures and rehabilitation training, to convey the benefits of sports activities to the older adult and stimulate interest in active participation. 3) Governments and communities can encourage the organization of social sports activities, such as older adult sports clubs and sports competitions, to enhance the social and recreational aspects of participation. 4) Advocate for community sports activities that involve all age groups, enabling the older adult to participate alongside people of other age groups, fostering intergenerational communication and interaction. 5) Establish health incentive programs to encourage older adult participation in sports activities, such as offering discounted gym memberships and health food shopping vouchers. Through these measures, we aim to increase the older adult’s engagement in sports activities, promote their health conditions, and contribute to the promotion of healthy aging through physical activities.

Second, active participation in social activities among older adults significantly improves their health status. The more diverse the types and forms of social activities they engage in, and the higher the frequency of participation, the more pronounced the effect on improving the health status of older adults, especially in alleviating cognitive depression [23]. The results indicate that older adults’ participation in social and recreational activities has a significant impact on their health status. By increasing their involvement in such activities, older adults can achieve a better state of mental health, including lower levels of depression and anxiety. Active social interaction may also help slow down cognitive decline: 1)The government could allocate funds to support social activity centers, facilitating the organization of social and recreational events such as dances and recreational activities. 2)Collaboration between the government and communities can involve hosting various cultural performances, such as concerts and theatrical productions, providing opportunities for the older adult to appreciate and participate in cultural activities. 3)Encourage the participation of older adults in volunteer services, such as community service and volunteer activities, fostering opportunities for social interaction and shared contributions. 4)Provide social skills training for the older adult to enhance their confidence and abilities in social interactions. 5)The government can offer training and support to encourage older adults to use digital social platforms, expanding their social networks and involvement in online communities.6) By constructing age-friendly communities and designing public spaces to promote social interaction, such as parks and leisure squares, a diverse and happy community for the older adult can be created. This aims to provide platforms for increased social and recreational activities, optimizing the late-life experience and enhancing overall well-being and health.

Third, Improving physical health and daily home activity abilities are closely associated with the social network of older adults [24]. Online activities significantly impact the health status of older adults. Participation in online activities may help alleviate loneliness and enhance happiness. Although online socialization differs from face-to-face interaction, it provides older adults with an alternative social channel, expanding their social circle and reducing social barriers. Engaging in online activities, such as learning new digital skills and participating in online games, may provide cognitive stimulation for older adults, contributing to maintaining brain agility: 1) Advocate for the implementation of digital training programs to assist older adults in acquiring basic digital skills, including using social media and email. 2) Enhance the quality of digital infrastructure to ensure that older individuals can easily access the internet, including providing free Wi-Fi coverage and financial assistance for device acquisition. 3) Encourage community organizations to organize online social activities, such as online social clubs and virtual forums, to meet the digital socialization needs of older adults. 4) Create a digital-friendly environment in communities, including establishing digital social corners and providing digital support services, to facilitate better engagement of older adults in online social activities. 5) Provide digital mental health resources, such as online counseling services, to support older adults in accessing mental health assistance through digital channels. Enhancing the online participation of older individuals helps them integrate better with society, gain insights into healthier lifestyles, and thereby promote their overall health and well-being.

Forth, the health of older adults is influenced by a combination of physical exercise, social and recreational activities, and online engagement. Good health status is closely associated with higher levels of physical activity. Living in areas with good recreational facilities and participating in social and recreational activities are significantly correlated with engaging in physical activities at recommended levels [25]. Policies focusing solely on one domain are insufficient to comprehensively address the diverse needs of older adults. By integrating policies and resources from different domains, a more comprehensive and effective approach can be adopted to promote the health of older adults: 1) Establish dedicated interdisciplinary task forces that bring together expertise from health, social, digital, and other relevant fields to collaboratively formulate and oversee the implementation of health policies for the older adult. 2) Create an interdisciplinary information-sharing platform to facilitate the exchange of data and research findings, ensuring decision-makers have access to comprehensive information for informed decision-making. 3) Provide sports facilities, social activity spaces, and digital learning resources concurrently in community centers to better serve the diverse needs of older individuals. 4) Conduct interdisciplinary training and educational activities to enhance the understanding of comprehensive health needs among professionals in the fields of health, social services, and digital technologies. 5) Encourage collaborative projects among institutions and organizations in health, social services, and digital domains to achieve policy synergy and optimize resource utilization. By clearly delineating the advantages of interdisciplinary collaboration, decision-makers can receive guidance to ensure the effective implementation of comprehensive health policies for older individuals. This approach promotes their holistic engagement in social activities, enhances physical health, mitigates issues related to older adult care, and alleviates societal pressures associated with aging.

## Conclusion

Efforts are being made globally to achieve the goal of healthy aging, with increasing attention to the health issues of the older adult. This paper conducts empirical research on the health status of older adults through various social participation modes, namely physical activities, social and recreational engagements, and online activities. The findings reveal a significant impact of physical activities, social and recreational engagements, and online activities on the health status of older individuals, demonstrating a positive correlation. Based on these findings, the paper proposes the following recommendations: 1) Promote older adult physical activities by creating an integrated exercise environment, 2) Expand the social network of the older adult by constructing diverse and structured happy communities, 3) Develop the online life of the older adult to help them integrate into the digital era, and 4) Establish cross-disciplinary collaboration for the overall health of the older adult.

## Limitations and prospects

Limitations: 1)The utilization of cross-sectional data from CGSS2021 provides information for a single time point, limiting the ability to track the long-term effects of participation methods. This approach may not fully capture the dynamic relationship between social engagement and the health status of the older adult. 2)Relying on self-reported health conditions introduces subjectivity and potential memory biases. The absence of objective health indicators and medical data may impact the accuracy of health assessments. 3)The classification of social participation methods is relatively simplistic, failing to comprehensively consider the diversity of various engagement activities. A more detailed and nuanced classification could enhance the precision in capturing the impact of different activities on health. 4)Due to variable limitations, the study may not have comprehensively considered potential confounding variables, such as income levels and education, leading to an oversight of other factors influencing the health of the older adult.

In future research, adopting a longitudinal study design to track changes in the social participation methods and health status of the older adult will provide a more comprehensive understanding of their dynamic relationship. Integrating objective health indicators, including physiological data and medical test results, along with considering multidimensional factors like mental health and quality of life, can enhance the accuracy of assessing the health status of the older adult.A more in-depth exploration of various types of social participation, including volunteer services, cultural activities, and social gatherings, will contribute to a detailed understanding of their differential impacts on the health of the older adult. Methodologically, incorporating more potential confounding variables, such as economic status and education level, will aid in a more comprehensive exploration of the impact of social engagement methods on older adult health. These prospects aim to enhance the scientific and practical aspects of the research, providing deeper guidance for older adult health policies and practices.
